# Successful use of cyclosporine as treatment for eosinophilic cystitis: a case report

**DOI:** 10.1186/s40413-016-0113-4

**Published:** 2016-07-08

**Authors:** Sohaib Aleem, Bharat Kumar, Mary Beth Fasano, Elizabeth Takacs, Antoine Emile Azar

**Affiliations:** Division of Immunology, University at Iowa, 200 Hawkins Drive, Iowa City, IA 52242 USA; Department of Urology, University at Iowa, 200 Hawkins Drive, Iowa City, IA 52242 USA; Present address: Division of Allergy and Clinical Immunology, John Hopkins Asthma & Allergy Center, 5501 Hopkins Bayview Circle, Baltimore, MD 21224 USA; Division of Immunology, Department of Internal Medicine - C42 GH, University of Iowa Carver College of Medicine, 200 Hawkins Drive, Iowa City, IA 52242 USA

**Keywords:** Eosinophilic cystitis, Cyclosporine, Steroids

## Abstract

**Background:**

Eosinophilic cystitis is a rare inflammatory disorder characterized by eosinophilic infiltration of all layers of the urinary bladder wall. Due to lack of consensus and potential for side effect from various therapeutic options, treatment of the disease is often challenging.

**Case presentation:**

A 64-year old woman with hypertensive nephropathy resulting in stage III chronic kidney disease, obstructive sleep apnea, and obstructive lung disease presented with a 4 month history of dysuria, urgency, frequency, and persistent hematuria. Based on eosinophilic infiltration on bladder wall biopsy in the absence of any evidence of infection, malignancy, or immune disorder, she was diagnosed with eosinophilic cystitis. Despite multiple medication regimens, her symptoms persisted, requiring high-dose prednisone with steroid-related side effects. After four months, she was started on cyclosporine, which led to symptomatic improvement and reduction in prednisone dosage. At that time, repeat urine cytology and cystoscopy did not reveal friable tissues or eosinophiluria.

**Conclusion:**

This case illustrates the utility of using cyclosporine to treat eosinophilic cystitis in adult patient with multiple comorbid conditions.

## Background

Eosinophilic cystitis (EC) is a rare inflammatory disorder characterized by eosinophilic infiltration of all layers of the urinary bladder wall, muscle necrosis, and fibrosis of the mucosa and muscularis propria [1]. Due to its rarity, not much is known about EC, and most of the data is derived from individual case reports and case series. Typically, patients with EC present with increased urinary frequency, nocturia, dysuria, hematuria, suprapubic pain and urinary retention [1–3]. Though there is a higher prevalence among adults, EC has been known to affect all age groups [1]. It has been associated with a number of other disorders, including chronic vesical injury, bladder neoplasms, interstitial cystitis, recurrent urinary tract infections, parasitic infections, atopic diseases and chronic granulomatous disease [2, 4–6]. Biopsy is essential for the diagnosis of EC, since other forms of cystitis may also be accompanied by eosinophiluria and cystoscopic changes that resemble EC [2, 3]. Despite multiple proposed systemic, surgical and intravesical approaches, treatment for the condition is often frustrating due to the lack of consensus and high rate of recurrence [1, 2, 7]. Among promising therapeutic agents, cyclosporine, a cyclophilin inhibitor whose downstream effects include reducing the serum levels of Interleukin-4 (IL-4), IL-5, and IL-13, has been used successfully as long-term treatment, as documented in two pediatric cases reports [5, 8]. We advance the medical literature by describing our experience of using cyclosporine in an adult female with biopsy-proven EC.

## Case presentation

A 64-year old female with chronic kidney disease stage III secondary to longstanding hypertension, chronic obstructive pulmonary disease, and obstructive sleep apnea presented for evaluation of dysuria, urgency, frequency, and persistent hematuria for 4 months at the University at Iowa Hospitals and Clinics in July 2014. The rest of the review of systems was unremarkable. She had no history of atopic disease, including allergic rhinitis, asthma, and atopic dermatitis, eosinophilia, parasitic infections, toxin or radiation exposures, or recent travel. Her medication list at initial appointment with immunology included albuterol, ciclesonide and salmeterol inhalers as well as theophylline XR 300 mg, solifnenacin 10 mg, phenazopyridine 100 mg, hydrocodone-acetamenophen 5–325 mg, nebivolol 2.5 mg, omeprazole 20 mg, sucralfate 1000 mg and multivitamin oral tablets/capsule. Methenamine mandelate was recently stopped by an outside urologist as it was thought to contribute to the symptoms. She was treated with several courses of antibiotics for urinary tract infections (UTIs) since the onset of symptoms. Vital signs were stable and physical examination revealed only mild suprapubic tenderness. Cystoscopy performed prior to the visit in July 2014 showed multiple friable mucosal lesions without tumor, stone, or clot, which was consistent with diffuse hemorrhagic cystitis. Her work up during the visit showed pyuria with hematuria, elevated inflammatory markers including erythrocyte sedimentation rate, C-reactive protein and leukocytosis with neutrophilia but no peripheral eosinophilia. She had elevated Interleukin-2R (CD25) and Th2 cytokines (Interleukin-4, Interleukin-5 and Interleukin-13) with elevated Immunoglobulin E (see Table 1).

**Table 1:** Cytokine panel and IgE level for 65 year old patient with eosinophilic cystitis

Tests	Reference range	Patient’s level
IgE	0–100 IU/mL	551
Soluble Interleukin-2 Receptor (CD25)	≤1033 pg/mL	1351
Interleukin-12	≤6 pg/mL	<5
Interferon-γ	≤5 pg/mL	12
Interleukin-4	≤5 pg/mL	8
Interleukin-5	≤5 pg/mL	7
Interleukin-10	≤18 pg/mL	10
Interleukin-13	≤5 pg/mL	232
Interleukin-1β	≤36 pg/mL	22
Interleukin-6	≤5 pg/mL	<5
Interleukin-8	≤5 pg/mL	<5
Tumor necrosis factor-α	≤22 pg/mL	36
Interleukin-2	≤12 pg/mL	12

**Fig. 1:**
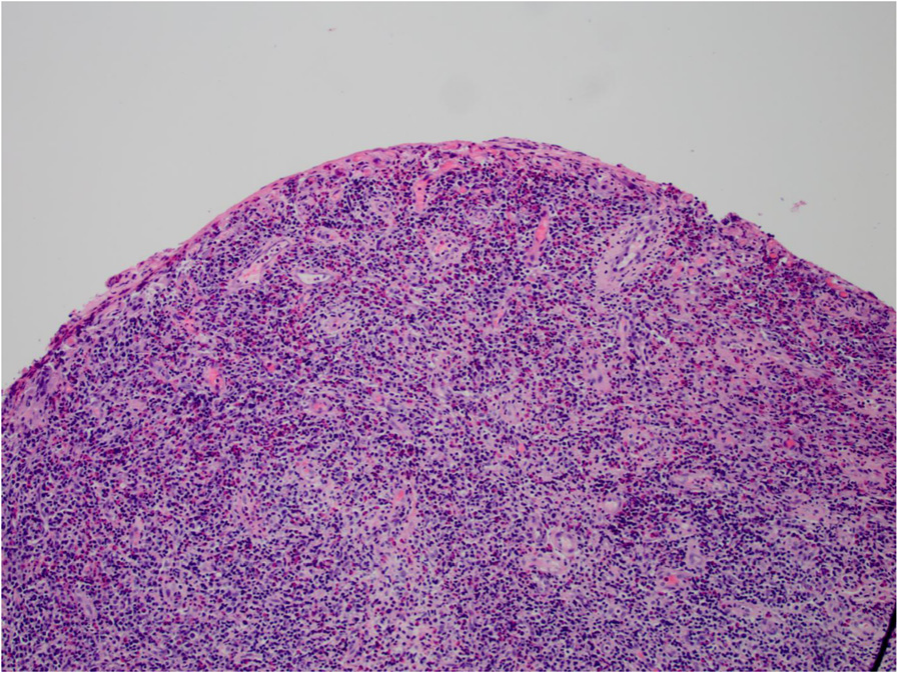
Low resolution hematoxylin and eosin (H&E) staining of bladder wall biopsy of the patient, showing abundant eosinophils in mucosa and submucosa

**Fig. 2:**
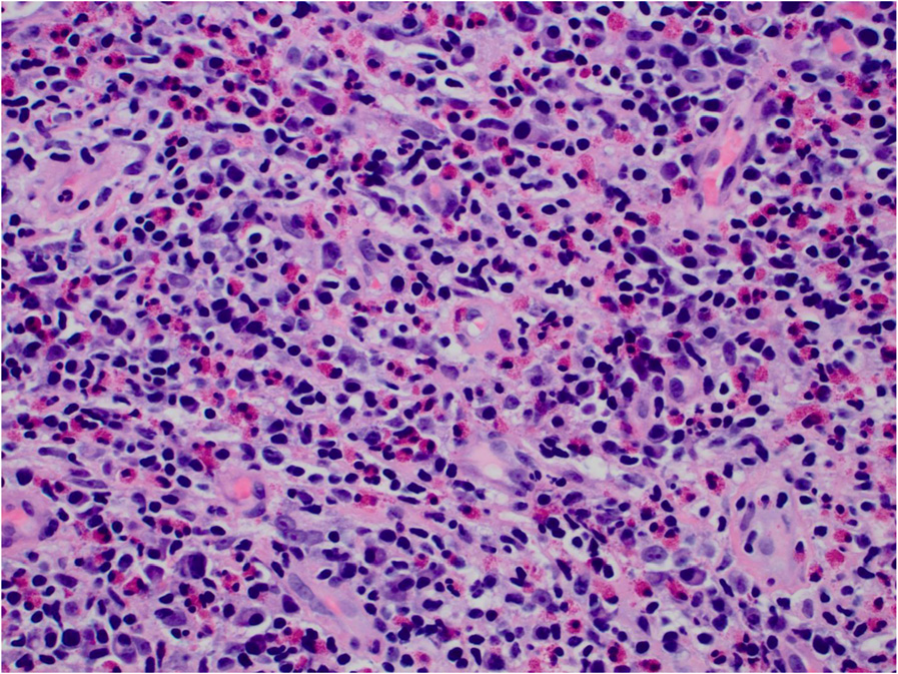
High resolution hematoxylin and eosin (H&E) staining of bladder wall biopsy of the patient, showing abundant eosinophils in submucosa

She had no serologic evidence of prevalent parasitic infections in the area (Strongyloides and Toxocara) or autoimmune disease. Cytology and culture from bladder washings in July 2014 did not identify any tumor cells or infection; biopsy of the bladder wall, which contained sections of the muscularis propria, showed eosinophil-rich inflammatory infiltrate (Figs. 1 and 2).

Gram- and acid-fast bacilli stains of the biopsy were also negative. Given the lack of tumor or infection, she was diagnosed with eosinophilic cystitis.

At first, she was given combination of oral hydroxyzine 30 mg three times daily, oral prednisone taper (40 mg, 30 mg 20 and 10 mg) with dose reduced every 5 days and solifenactin without significant improvement in symptoms and hematuria. Later, she was given a trial of 6 weekly courses of intravesical methylprednisolone followed by oral prednisone, addition of loratadine 10 mg daily and monteleukast 10 mg daily with partial improvement in her symptoms without resolution of hematuria. Symptoms and hematuria would worsen if the dose of prednisone was reduced below 25 mg daily. Additionally, she developed several side effects of chronic prednisone use, including tremors, weight gain, depressed mood, visual changes, and a fragility fracture of the hip, which mandated significant dose reduction and resulted in worsening of her symptoms.

Repeat cystoscopy in November 2014 showed areas of friable tissue similar to July 2014; urodynamic study in December 2014 showed small capacity bladder suggesting fibrosis of bladder wall as a complication of EC resulting in Grade 2 bilateral vesicoureteral reflux and higher risk of recurrent UTIs. She was offered surgical treatment options including suprapubic catheter, cystectomy or urinary diversion for relief of symptoms which she declined. After weighing the risks and benefits of different therapy options, oral cyclosporine (50 mg twice a day) was added to her regimen for a steroid-sparing effect and decrease Th2 cytokine profile. Over the course of three months, her prednisone dose was decreased to 7.5 mg daily, which helped to reduce her depression, tremors, and weight gain. Her renal function, as well as blood pressure, remained stable. Despite being on a potent immunosuppressant, her renal function as well as blood pressure remained stable and frequency of recurrent UTIs did not increase compared to July 2014. Due to side effects of nausea and recurrence of tremors with two weeks trial of increased dose of cyclosporine 75 mg twice daily, further dose escalation of cyclosporine were avoided. Repeat urine cytology in April 2015 did not show any eosinophils or tumor cells. She had a repeat cystoscopy after 6 months of cyclosporine use that did not show any friable mucosa, therefore no biopsy was done. Moreover, she relayed decreased urgency and frequency, and resolution of her overt hematuria.

## Discussion

To our knowledge, this is the first case to document cyclosporine as treatment for EC in an adult patient with multiple comorbidities. Although cyclosporine did not reverse the fibrosis of the bladder wall secondary to EC, it allowed reduction in dose of oral steroids and lead to sustained significant improvement in the patient’s symptoms without major adverse effects. Serum cytokine profile was consistent with Th2 polarization; however eosinophilia was absent in the patient. This observation is in line with our current knowledge of how cyclosporine works: as a cyclophilin inhibitor, it inhibits interleukin-2 gene transcription and thus prevents T-cell proliferation and Th2 polarization. Even though long term safety data about use of cyclosporine in EC is not available, it has been well tolerated when used for interstitial cystitis [9]. Further studies are needed to evaluate the efficacy and safety of cyclosporine use for EC in adult patients with multiple comorbid conditions.

## Conclusion

This case report illustrates the clinical utility of using cyclosporine to treat eosinophilic cystitis in adult patient with multiple comorbid conditions.

## Abbreviations

EC, Eosinophilic cystitis; Th2, T helper cell 2; UTIs, Urinary tract infections
